# Clinical Outcomes After Intramedullary Nailing of Intraarticular Distal Tibial Fractures: A Retrospective Review

**DOI:** 10.5435/JAAOSGlobal-D-20-00088

**Published:** 2020-06-10

**Authors:** Kathryn B. Metcalf, Corina C. Brown, Edward M. Barksdale, Robert J. Wetzel, John K. Sontich, George Ochenjele

**Affiliations:** From the Department of Orthopaedic Trauma Surgery, University Hospitals Cleveland Medical Center, Case Western Reserve University School of Medicine, Cleveland, OH.

## Abstract

The objective of this study was to evaluate the clinical and functional outcomes of intra-articular distal tibial fractures after intramedullary nail (IMN) and independent fixation compared with extra-articular fracture controls.

**Methods:**

A retrospective chart review of distal tibial fractures treated with IMN was performed. Clinical outcomes were compared between fractures with and without intra-articular involvement. Outcomes included nonunion, malunion, ankle arthrosis, and infection. Patient-Reported Outcome Measurement System (PROMIS) scores were used to assess subjective outcomes.

**Results:**

Of the 135 distal tibial fractures, 87 extra-articular and 48 intra-articular, no significant difference was observed in the rate of ankle arthrosis between intra-articular and extra-articular fractures (2% versus 0%; *P* = 0.35). Similarly, no difference was observed in the postoperative rates of infection (8% versus 3%; *P* = 0.25), the rate of nonunion (17% versus 10%; *P* = 0.29), or the rate of malunion (10% versus 21%; *P* = 0.17). No notable difference was observed in PROMIS scores between groups.

**Conclusion:**

This study suggests that IMN is an acceptable method of fixation in select intra-articular distal tibial fractures. In the intra-articular group, low rates of ankle arthrosis were noted at intermediate follow-up, with no increase in nonunion, malunion, or infection compared with extra-articular fractures. Furthermore, PROMIS scores indicate similar functional outcomes in patients, regardless of intra-articular involvement.

Distal tibial fractures occur because of both high- and low-energy mechanisms. They can present challenges in healing with relatively high rates of nonunion and malalignment despite fixation^1^ and further complicating the management of distal tibial fractures is the potential for intra-articular extension. This can occur from both axial and torsional forces, resulting in pilon fractures (complex) and fractures with large posterior malleolar (simple) components.^2,3^ However, it is difficult to differentiate these types of fractures in the literature, and there is a paucity of studies that compare treatment and clinical outcomes. Thus, the optimal treatment of distal tibial fractures, particularly those with intra-articular extension, remains debatable.^4-6^

Because of the benefits of intramedullary nail (IMN) fixation for tibial shaft fractures, including minimizing soft-tissue disruption, the use of IMN in the treatment of distal tibial fractures has increased. This is true of both extra-articular and intra-articular distal tibial fractures. Although open reduction and internal fixation of intra-articular distal tibial fractures is the traditional choice and still common, the alternative of IMN fixation offers a lower risk of soft-tissue complications and potentially earlier definitive fixation when percutaneous techniques are used.^7^ Previous reports suggest that an IMN fixation method with additional independent fixation of intra-articular fragments is a safe method of fixation.^7,8^ Furthermore, it has been reported in a biomechanical study that while no difference was observed in overall biomechanical stability with torsional forces, IMN fixation of intra-articular distal tibial fractures has superior axial loading compared with medial plate fixation.^9^ Although IMN fixation of intra-articular distal tibial fractures is potentially more technically difficult than intramedullary nailing of extra-articular fractures, the advantages of IMN fixation of intra-articular fractures is appealing. However, studies that compare outcomes of intra-articular and extra-articular distal tibial fractures treated with IMN fixation are limited. No study to the authors' knowledge has evaluated the combination of both radiographic and clinical outcomes.

The objective of the current study was to evaluate and compare the outcomes of intra-articular distal tibial fractures after intramedullary fixation compared with extra-articular fractures. The authors predicted that there would be a higher incidence of ankle arthrosis in fractures with joint involvement. Furthermore, because of the complexity of these injuries and the more extensive fixation, we expected that the distal tibial intra-articular fractures would be associated with higher rates of nonunion, malunion, and infection. Finally, we hypothesized that patient-reported outcomes would be more favorable in patients with extra-articular distal tibial fractures treated with IMN fixation.

## Methods

**Figure 1:**
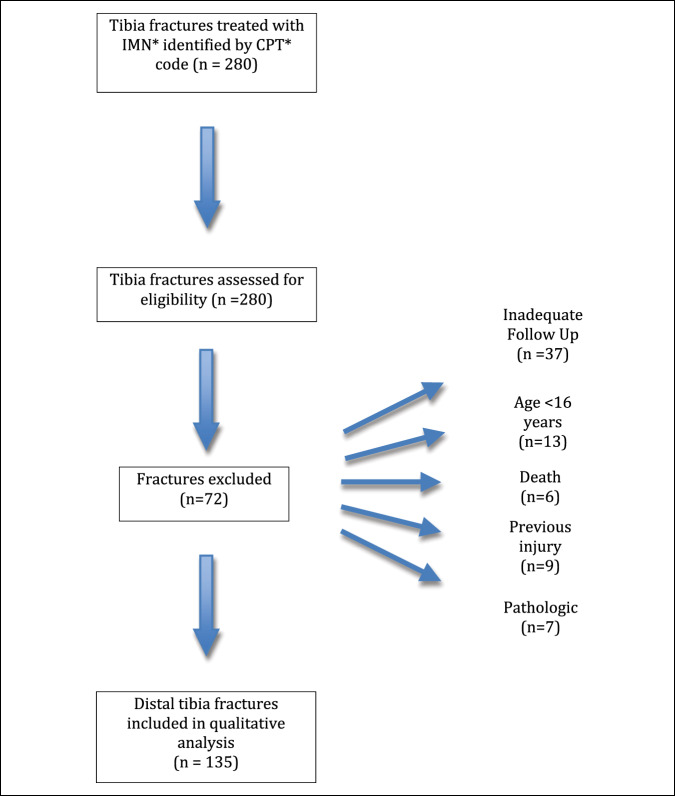
Chart demonstrating the patient inclusion. CPT = current procedural terminology, IMN = intramedullary nail

A retrospective review was performed on patients who sustained tibial fractures treated with intramedullary fixation at a Level I trauma center between 2008 and 2018. Current procedural terminology codes were used to establish the initial cohort. Two hundred seventy-six (276) patients were identified with 280 tibial fractures. Medical charts were reviewed for demographic information, mechanism of injury, fracture type, and postoperative complications (Table 1). Inclusion criteria were met if the patient sustained a distal tibial fracture treated with an IMN, was 16 years of age or greater, and had follow-up until clinical and radiographic union and full weight-bearing or longer. A total of 72 subjects were excluded for lack of follow-up to time of union and full weight-bearing (37), pathologic fracture (7), previous injury or surgery to injured tibial or ankle (9), greater than 16 years of age (13), or death before follow-up (6) (Figure 1). Patients had a mean time for follow-up of 9 months in the extra-articular group and 10 months in the intra-articular group. Patients were included with less than 6 months of follow-up if they had both clinical and radiographic evidence of union as the final follow-up.

**Table 1:** Distal Tibial Fracture Demographics and Mechanism of Injury

Fracture	Demographics		Trauma	Mechanism						
	Average Age	Sex (M)	Polytrauma	MVC	Pedestrian	Fall From Height	GSW	Ground Level Fall	Sports	Other
Distal tibial fractures	45.3 (16–84)	55%	8% (n = 12)	17% (n = 23)	5% (n = 7)	18% (n = 24)	7% (n = 10)	29% (n = 39)	7% (n = 9)	17% (n = 23)

MVC = motor vehicle collision, GSW = gunshot.

**Figure 2:**
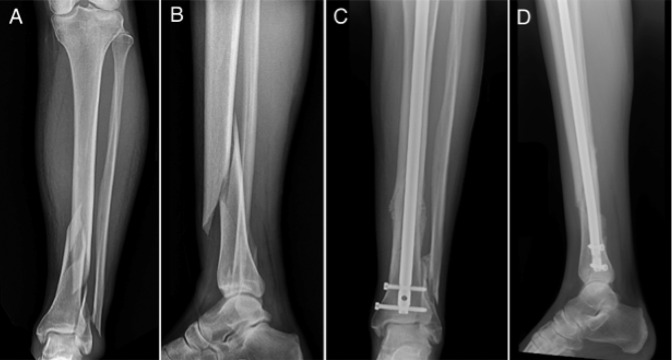
Radiograph demonstrating the (**A**) AP and (**B**) lateral injuries of an extra-articular distal tibial fracture and (**C**) AP and (**D**) lateral radiographs following intramedullary nail fixation.

**Figure 3:**
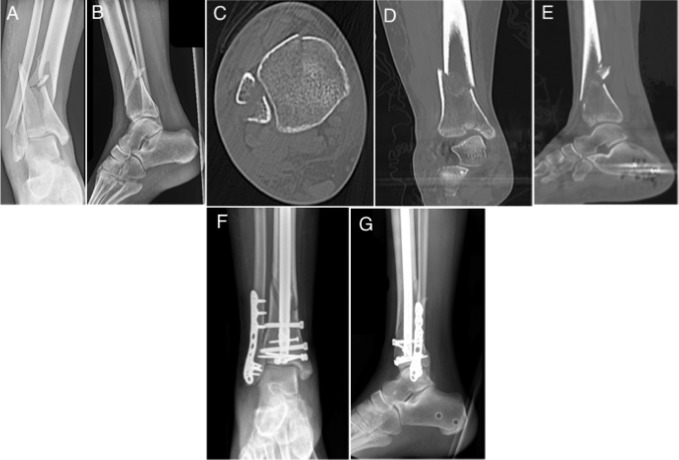
Radiograph demonstrating the (**A**) AP and (**B**) lateral injuries and (**C**–**E**) injury CT images of an intra-articular distal tibial fracture. Postoperative (**F**) AP and (**G**) lateral radiographs after intramedullary nail and screw fixation of the articular fracture.

**Figure 4:**
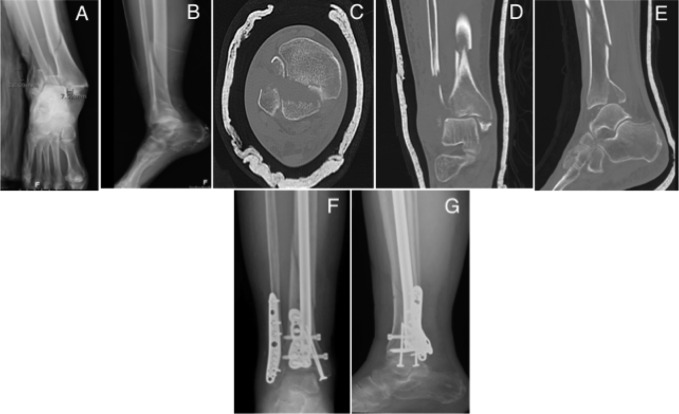
Radiograph demonstrating the (**A**) AP and (**B**) lateral injuries of a distal tibial fracture with intra-articular extension into the plafond. CT images (**C**) axial (**D**) coronal and (**E**) sagittal of the injury. Radiographs (**F**) AP and (**G**) lateral at 6 weeks postoperative fixation with IMN and plate fixation of intra-articular fracture with maintained alignment.

**Figure 5:**
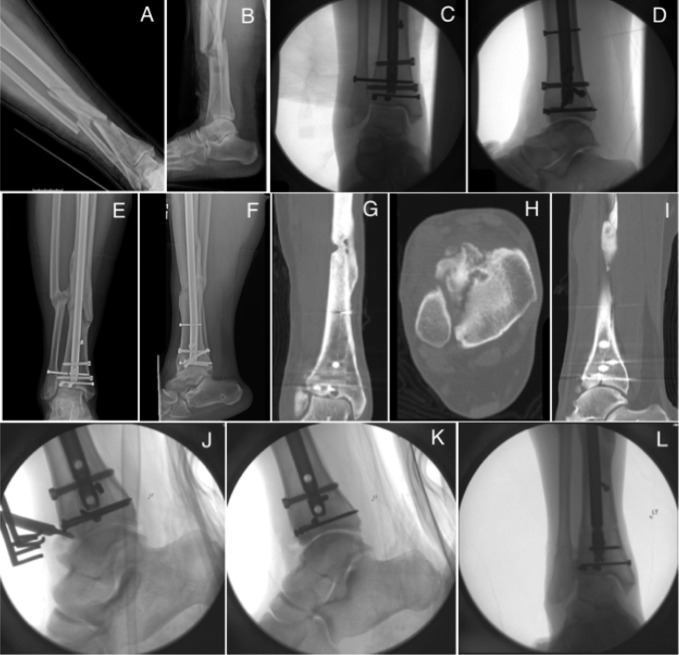
Radiograph demonstrating the (**A**) AP and (**B**) lateral injuries of initial injury and subsequent (**C**) AP and (**D**) lateral intraoperative fluoroscopic images of initial fixation of an intra-articular distal tibial fracture. Radiographs (**E**) AP and (**F**) lateral and CT (**G**) axial, (**H**) coronal, and (**I**) sagittal images after IMN and screw fixation that resulted in a single case of ankle arthrosis. The patient was treated with screw removal and débridement with removal of the anterior osteophyte (**J**–**L**).

Tibial fractures were considered distal third if they involved the distal third, were distal to the isthmus of the tibia, and were within 10 cm of the joint line.^6,10^ Distal tibial fractures were classified into the following two groups: extra-articular or intra-articular, determined with CT. Extra-articular distal tibial fractures were compared with fractures with intra-articular involvement (Figures 2 and 3). Fractures with displacement of the articular surface were treated with additional fixation independent of intramedullary nailing, which included either lag screw fixation or mini-fragment plate fixation, or both (Figures 3–5). Of the fractures with joint involvement, 11 were treated with staged fixation with temporary stabilization with an external fixator before definitive fixation on average 7.5 ± 6.3 days after injury. By contrast, only four extra-articular distal tibial fractures were temporarily stabilized. Lag screw fixation of the intra-articular fractures was used in most cases (35) compared with plate fixation (1) or both (3). Nine distal tibial fractures with intra-articular involvement were treated without independent lag screw or plate fixation because the articular components in these fractures were simple non-displaced posterior malleolus fractures at non–weight-bearing surfaces. None of these nine fractures had secondary displacement with nail insertion.

All IMNs were performed through an infrapatellar or suprapatellar approach. In the intra-articular group, there were 31 suprapatellar and 17 infrapatellar nails, versus the extra-articular group with 32 suprapatellar and 55 infrapatellar nails. All patients with intra-articular distal tibial fractures were made non–weight-bearing initially for an average of 8.4 ± 3.6 weeks. Of patients with extra-articular distal tibial fractures, 10 were made immediately weight-bearing, whereas 6 were partial weight-bearing, and 71 were non–weight-bearing. Non–weight-bearing of extra-articular fractures became progressively less in fractures treated in more recent years. The average time for non–weight-bearing of extra-articular distal tibial fractures was 5.8 ± 2.9 weeks.

Outcome measurements included ankle arthrosis, fracture nonunion, fracture malalignment, and postoperative infection. Arthrosis was diagnosed by radiographs and confirmed by CT in addition to clinical symptoms of ankle pain and stiffness. Union was defined as radiographic evidence of three bridging cortices of bone and asymptomatic full weight-bearing at the final follow-up. Malalignment was defined as ≥5° of angulation in any plane.^11^ Radiographic alignment and arthrosis were measured by the senior author (G.O.), who is a fellowship-trained orthopaedic trauma surgeon. Infection was determined if there was presence of skin necrosis, purulent drainage, systemic signs of infection, elevated inflammatory markers, abscess formation, or imaging consistent with a fluid collection. Two patients with combined malalignment and nonunion underwent further surgical treatment including nail dynamization in one case and open reduction and internal fixation in the other. All other cases of malalignment were managed conservatively based on symptoms.

Subjective outcomes were measured retrospectively by patient-reported knee pain and Patient-Reported Outcome Measurement System (PROMIS) physical function (PF) and pain interference (PI). These scores are standardized based on a scale of 0 to 100 with a mean of 50 and SD of 10, which represents the general US population. Higher PF scores indicate greater PF, whereas higher PI scores represent greater pain limiting physical, mental, and social activities of the patient. This has been validated as a subjective outcome measurement in lower extremity trauma.^12^ The PI and PF scores were collected either in the clinic or via a telephone interview. Outcomes were compared between distal tibial fractures with and without intra-articular extension. These were measured at the patients' final follow-up in both groups. The average time to PROMIS score collection at the final follow-up was 9 months.

The rate of ankle arthrosis, nonunion, malunion, and infection were compared between groups. Data analysis of these outcomes was completed using Fischer Exact and Chi-squared tests where indicated. To compare the mean PF and PI outcome measures, statistical analysis included Student *t*-tests where appropriate. Statistical significance was set at *P* ≤ 0.05.

## Results

A total of 135 distal tibial fractures that met the inclusion criteria were identified. Of these patients, 55% (n = 75 of 135) were men and 45% (n = 60 of 135) women with an average age of 45.3 years, ranging from 16 to 84 years. All distal tibial fractures (n = 135 of 135) underwent IMN fixation with a suprapatellar or infrapatellar approach. Thirty-six percent (n = 48 of 135) of fractures demonstrated intra-articular involvement. Eighty-one percent of intra-articular fractures (n = 39 of 48) were treated with additional fixation. There was an average follow-up time of 9 months for the extra-articular group and 10 months for the intra-articular group.

The rate of ankle arthrosis in intra-articular fractures was 2% (n = 1 of 48) compared with none in extra-articular fractures (*P* = 0.35) (Figure 5). There was a 17% (n = 8 of 48) incidence of nonunion associated with intra-articular fractures compared with the nonunion incidence of 10% (n = 9 of 87) in fractures without joint involvement (Table 2). No significant difference was observed in nonunion rates between intra- and extra-articular fractures (*P* = 0.29).

**Table 2:** Incidence of Complications in Extra-articular Versus Intra-articular Distal Tibial Fractures

Fracture Type	Total	Infection	*P*	Nonunion	*P*	Malunion	*P*	Arthrosis	*P*
Extra-articular fracture	87	4% (n = 3)		10% (n = 9)		21% (n = 18)		0%	
Intra-articular fracture	48	8% (n = 4)	0.25	17% (n = 8)	0.29	10% (n = 5)	0.17	2% (n = 1)	0.35

Similarly, no difference was observed in the rate of infection between the two groups (*P* = 0.25). Intra-articular fractures demonstrated an 8% (n = 4 of 48) infection rate, whereas extra-articular fractures had a 3% (n = 3 of 87) incidence of infection postoperatively. This was true despite the higher percentage of open fractures in intra-articular fractures. More open injuries occurred in fractures that involved the joint 33% (n = 16 of 48) compared with extra-articular fractures 22% (n = 19 of 87), but this difference was not significant (*P* = 0.14).

Malunion was compared between fractures with and without intra-articular involvement. No significant difference was observed in the incidence of malunion in distal tibial fractures with joint involvement compared with extra-articular distal tibial fractures (10% versus 21%; *P* = 0.17) (Table 2). Almost all fractures with intra-articular involvement were treated with lag screw fixation of the articular component, followed by IMN of the metaphyseal fracture component (Figure 5). Patients with intra-articular fractures were more likely to have fibular fixation compared with extra-articular fractures (33% versus 16%; *P* = 0.02). Fibular fixation did not confer any significant improvement in alignment, compared with no fibular fixation (*P* = 0.17). In addition, the intra-articular fractures were treated using a suprapatellar approach in 65% of cases, whereas an infrapatellar approach was used in 63% of extra-articular fractures. Of extra-articular fractures that resulted in malunion, 78% (n = 14 of 18) were treated with an infrapatellar approach. The overall difference in malunion between distal tibial fractures treated with a suprapatellar versus infrapatellar approach was significantly different (*P* = 0.02).

The PROMIS scores, both PI and PF, were evaluated across groups. The average PI score for the extra-articular group was 55.1 ± 7.8, whereas the average of the intra-articular group was 59.4 ± 9.3 (Table 3). Although the PI score of intra-articular fracture was slightly higher, no statistically significant difference was observed between PI scores across the two groups (*P* = 0.14). No significant difference was observed in PF scores in the extra-articular versus intra-articular fracture groups with averages of 41.9 ± 7.6 and 42.3 ± 8.4, respectively (*P* = 0.87). Malunion and nonunion did not seem to have a notable effect on PI or PF scores. In the extra-articular group, 57% (n = 12 of 21) reported anterior knee pain compared with the 71% (n = 12 of 17) of patients in the intra-articular group who reported anterior knee pain. This was not significantly different (*P* = 0.22).

**Table 3:** PROMIS Score Comparison

Fracture	PI PROMIS	*P*	PF PROMIS	*P*
Extra-articular	55.1 ± 7.8		41.9 ± 7.6	
Intra-articular	59.4 ± 9.3	0.14	42.3 ± 8.4	0.87

PF = physical function, PI = pain interference, PROMIS = Patient-reported Outcome Measurement System

## Discussion

The objective of this review was to evaluate the clinical outcomes of intra-articular distal tibial fractures compared with extra-articular distal tibial fractures treated with intramedullary nailing. Despite the complex nature of distal tibial fractures, there seems to be no notable difference in many outcome measurements in this study between intra-articular and extra-articular fractures after intramedullary nailing. The intra-articular distal tibial fractures were more complex injuries. Independent fixation of the articular segment and intramedullary fixation of the metadiaphyseal component was performed. The incidence of nonunion and infection was not notably different between the two groups. The rates of nonunion found in this study are similar to the previously described rates of nonunion in tibial fractures treated with IMN.^1,13,14^ In addition, the incidence of infection is comparable with previous reports of tibial fractures treated with IMN fixation.^15^ However, it is important to recognize that there are limited studies evaluating these outcomes in intra-articular distal tibial fractures after intramedullary nailing, and although this is a small cohort, it is one of the largest presented in the literature.

The lack of a notable difference in nonunion between groups was not expected. We anticipated that the intra-articular fracture group would have a prolonged period of restricted weight-bearing compared with the extra-articular group. Previous studies that have evaluated the nonunion rate of other long bone fractures with associated ipsilateral injuries that limits immediate weight-bearing, such as femoral shaft fractures with a concomitant femoral neck fracture, have shown greater rates of nonunion.^16^ Potential explanations for such results were the early restrictions in weight-bearing. In this study, many distal tibial fractures, both intra-articular and extra-articular, were treated with restricted weight-bearing, primarily because of the distal nature of the fracture.

Interestingly, there seems to be a higher rate of open injuries in fractures that involved the joint. However, this was only a trend, and no notable difference between fractures with and without intra-articular involvement was appreciated. Potentially higher energy mechanisms created intra-articular extension of fractures in addition to causing open injuries. The fixation strategy of using combined either percutaneous or limited open approach to fix the joint and IMN for metadiaphyseal fixation seems to be effective in minimizing soft-tissue complication in this group of complex injuries because no difference in infection was observed between groups.

There was only one case of ankle arthrosis in an intra-articular distal tibial fracture, which was not notably different compared with extra-articular fractures. In this single case of ankle arthrosis, the patient was noncompliant with weight-bearing restrictions postoperatively (Figure 5, E–I). This could have contributed to her ankle arthrosis. However, her fracture was a high energy displaced intra-articular fracture in which articular impaction and chondral injury potentially occurred despite anatomic radiographic reduction (Figure 5, A–D). The patient subsequently underwent distal interlock screw removal and débridement of the ankle (Figure 5, J–L). The patient has not required any additional surgical intervention. Overall, the rate of ankle arthrosis was not different between groups as expected. This suggests that IMN fixation with supplementary articular fixation in select intra-articular distal tibial fractures is an acceptable strategy. However, it is important to note that most of the fractures demonstrated a simple articular pattern, and anatomic reduction was achieved before nailing.

Malunion was lower in the intra-articular group, but not statistically notable. A precise, anatomic reduction of the joint surface was achieved when there is intra-articular involvement. Although these were more complex injuries than the extra-articular fractures, the meticulous reduction and fixation achieved at the articular surfaces potentially provided indirect metadiaphyseal reduction. Furthermore, there was a notable increase in fibular fixation in the intra-articular fracture group compared with extra-articular fractures. As previous studies have reported improved alignment and stability after fibular fixation in addition to IMN of distal tibial fractures, this potentially contributed to the lower malunion rate intra-articular fractures.^17,18^

The 21% rate of malunion in extra-articular fractures was high, but within the range reported in the literature after IMN of distal tibial fractures.^19^ The lack of fibular fixation in many extra-articular fractures could, in part, account for the higher malalignment.^17,18^ Furthermore, compared with intra-articular fractures, there were a greater number of extra-articular fractures that underwent IMN fixation with an infrapatellar approach. The suprapatellar approach showed a notably lower malunion rate, and this approach was used more frequently in intra-articular fractures.^11^ Of note, notably more malunions resulted in extra-articular fractures treated with an infrapatellar approach compared with a suprapatellar approach. This potentially contributed to the higher rate of malunion because previous studies have suggested improved alignment of distal tibial fractures using a suprapatellar approach.^11^

Another factor considered was the weight-bearing status. All intra-articular fractures were made non–weight-bearing postoperatively as were many of the extra-articular fractures because of their distal nature. Not surprisingly, within the data set, in more recent years, restricted weight-bearing of extra-articular fractures became less common. Regardless, there seems to be similar complications between weight-bearing and non–weight-bearing extra-articular distal tibial fractures, which is consistent with the literature.^20^

Regarding subjective outcomes, no difference existed in PI or PF PROMIS scores in fractures with and without intra-articular extension. These results suggest that separate independent fixation of tibial fractures that extend into the plafond or posterior malleolus does not introduce inferior subjective outcomes. This is consistent with previous reports of subjective outcome measures; however, PROMIS PI and PF scores in tibial fractures treated with concomitant IMN fixation with independent intra-articular fixation compared with extra-articular tibial fractures have not been reported in the literature. It is likely that the low PROMIS PF scores are more related to the overall health and function of the patient and not necessarily isolated to the function of the tibia.

To the authors knowledge, this is the first study to evaluate the outcome measures of union and malalignment in addition to the clinical subjective patient-reported outcomes through PROMIS scores after intramedullary nailing of distal tibial fractures and provide a comparison between intra- and extra-articular fractures. However, in previous studies that have evaluated outcomes following the combination of individual fixation of the intra-articular component and intramedullary fixation of the tibial shaft have shown varying results. Often studies have had inconsistent methods of fixation, lacked specific outcome measures, or lacked comparison with a control group. In one study evaluating intramedullary nailing of distal tibial fractures with intra-articular extension, the authors found no clinical or radiographic complications in the presence of malleolar fractures compared with extra-articular fractures.^21^ However, the authors reported that malleolar fractures were only fixed if there was radiographic evidence of increased displacement after the insertion of the nail. Similarly, Konrath et al^5^ described distal tibial fractures with intra-articular extension where fixation of the intra-articular component occurred before nailing with no associated increase in complication rate reported. Although no increase in complications was observed, neither study assessed specific patient outcome measures, and inconsistency in independent fixation of the intra-articular fractures was present. Other studies describe fixation of intra-articular components followed by IMN as safe and efficacious for soft-tissue preservation but do not provide an outcome comparison with extra-articular distal tibial fractures.^6,10,22-24^

The results of the current study suggest that IMN fixation of intra-articular distal tibial fractures is not only safe but an acceptable method of fixation when compared with a control of extra-articular distal tibial fractures. These results are encouraging because intramedullary fixation of intra-articular distal tibial fractures allows for less disruption of the soft-tissues and applies the benefits of nail fixation. Many of the intra-articular fractures were treated with percutaneous lag screw fixation followed by IMN. Potentially, this provides improved soft-tissue management compared with an open technique or even a minimally invasive plate osteosynthesis technique. This is particularly important in more complex injuries and open injuries. Furthermore, the exceedingly low rate of ankle arthrosis illustrates that the independent additional fixation is an appropriate option in distal tibial fractures with intra-articular extension. Long-term follow-up to ensure this low rate of arthrosis continuing with time would be beneficial.

This study is not without limitations. A multitude of factors exists that may have influenced these outcome measures. Although some of these limitations were corrected for, such as open injuries, others were not. There were multiple different surgeons performing fixation of distal tibial fractures over the 10-year period. Of note, intra-articular fractures were primarily treated by fellowship-trained traumatologists. However, this was not true of all distal tibial fractures treated with intramedullary fixation. Surgeons treated tibial fractures with both external fixation and delayed definitive fixation versus immediate definitive management. Determination of intramedullary fixation was at the discretion of the surgeon. Therefore, primarily fractures with simple intra-articular extension were treated with intramedullary nailing and separate fixation of the joint. Over the 10-year period of this retrospective review, there was likely an increase in the intramedullary nailing of these distal tibial fractures with intra-articular involvement and the complexity of the intra-articular portion deemed appropriate for nail fixation. In addition, different approaches, both suprapatellar and infrapatellar, were used. More recently, it has been suggested that these two approaches have differences in the malunion rate.^11^ The postoperative protocol, including weight-bearing status differed among surgeons. Furthermore, because of the strict inclusion/exclusion criteria used, the sample size was limited during the review process. Evidently, further studies with larger cohorts and prospective studies evaluating these fractures after intramedullary fixation would be beneficial.

## Conclusion

This study suggests that IMN fixation of intra-articular distal tibial fractures is an efficacious option with the appropriate indications. There is no notable increase in nonunion, malunion, infection, or ankle arthrosis when intra-articular distal tibial fractures are treated with IMN fixation and separate independent fixation of the intra-articular block. Furthermore, PROMIS scores indicate that patients do not have inferior outcomes when treated with IMN and independent fixation of intra-articular distal tibial fractures.
